# Molecular characterization and tissue distribution of carnitine palmitoyltransferases in Chinese mitten crab *Eriocheir sinensis* and the effect of dietary fish oil replacement on their expression in the hepatopancreas

**DOI:** 10.1371/journal.pone.0201324

**Published:** 2018-08-01

**Authors:** Li Liu, Xiaowen Long, Deng Deng, Yongxu Cheng, Xugan Wu

**Affiliations:** 1 Centre for Research on Environmental Ecology and Fish Nutrition of the Ministry of Agriculture, Shanghai Ocean University, Shanghai, China; 2 Key Laboratory of Freshwater Aquatic Genetic Resources, Ministry of Agriculture, Shanghai Ocean University, Shanghai, China; 3 Shenzhen Alpha Feed Co. Ltd., Guangdong Shenzhen, China; 4 National Demonstration Centre for Experimental Fisheries Science Education, Shanghai Ocean University, Shanghai, China; 5 Shanghai Collaborative Innovation Centre for Aquatic Animal Genetics and Breeding, Shanghai Ocean University, Shanghai, China; University of Illinois, UNITED STATES

## Abstract

The carnitine palmitoyltransferase (CPT) family includes CPT 1 and CPT 2 that transport long-chain fatty acids into the mitochondrial compartment for β-oxidation. In this study, three isoforms (CPT 1α, CPT 1β and CPT 2) of the CPT family were cloned from Chinese mitten crab (*Eriocheir sinensis*) and their complete coding sequences (CDS) were obtained. Sequence analysis revealed deduced amino acid sequences of 915, 775 and 683 amino acids, respectively. Gene expression analysis revealed a broad tissue distribution for all three isoforms, with high CPT 1α and CPT 2 mRNA levels in the hepatopancreas of males and females. In males, CPT 1β was highly expressed in gill, heart, brain ganglia and muscle, while in females, CPT 1β-mRNA levels were relatively high in muscle, hepatopancreas and ovary tissue. The effects of dietary fish oil replacement on the expression of the three CPT isoforms in the hepatopancreas during gonadal development were investigated using five experimental diets formulated with replacement of 0, 25, 50, 75 and 100% fish oil by 1:1 rapeseed oil: soybean oil. The results showed that Diets 2# and 5# yielded higher CPT 1α and CPT 2 mRNA expression in males (*P* < 0.05), while in females, expression of all three CPT isoforms increased then declined in the hepatopancreas with increasing dietary fish oil replacement. The observed changes in CPT gene expression varied in different isoforms and gender, suggesting the three CPT genes might play different roles in fatty acid β-oxidation in *E*. *sinensis*.

## Introduction

Mitochondrial β-oxidation of fatty acids is central to the provision of energy in most organisms, but long-chain fatty acids (LC-FA) cannot cross the mitochondrial inner membrane directly in animals [1, 2]. Carnitine palmitoyltransferase 1 (CPT 1) and CPT 2 form a mitochondrial carnitine palmitoyltransferase system that mediates the uptake of LC-FA into mitochondria and plays an important role in the control and regulation of mitochondrial β-oxidation [3, 4]. CPT 1 (EC 2.3.1.21), located in the outer membranes of mitochondria, catalyses the carnitine-dependent esterification of coenzyme A to form acylcarnitine, and is a rate-limiting enzyme in fatty acid oxidation [5, 6]. CPT 2, located on the inner mitochondrial membrane, catalyses a reconversion to generate acyl-CoA and carnitine within the mitochondrial matrix, and carnitine acylcarnitine translocase then transfers acylcarnitine from the outer to the inner mitochondrial membrane [7].

In mammals, the CPT family is composed of four different isoforms encoded by distinct genes: a liver isoform (CPT 1α or L-CPT 1), a muscle isoform (CPT1β or M-CPT1), a brain isoform (CPT 10γ) and CPT 2 [8–11]. The tissue distribution of the three CPT 1 isoforms varies, but CPT2 enzymatic activity is found in all tissues. In general, CPT 1α is widely expressed in most tissues including liver, intestine and heart, while CPT 1β is mainly expressed in muscle, adipose tissue and heart. CPT 1γ expression is restricted to the central nervous system where it functions in the control of whole-body glucose homeostasis [3, 9, 12].

Gene expression and enzyme activity of CPT family members varies between different tissues, which suggests that the β-oxidation of fatty acid is a complex pathway in mammals, and activity of CPT enzymes is controlled by various regulatory mechanisms [13, 14]. A wide range of factors, such as fatty acid profile, starvation, over-feeding, environmental conditions, and developmental stage can affect CPT gene expression, resulting in changes in lipid metabolism in animals [15–20].

Abnormal fat deposition in farmed fish is a significant problem for the development of the aquaculture industry, which may be related to over-feeding [21], imbalance of feed nutrition [17] and the replacement of fish oil with vegetable oil in feed [22, 23]. Thus, research on the expression and physiological functions of CPT genes in aquatic animals may be helpful for understanding fat deposition in aquatic animals. To date, CPT 1 has been identified in several aquatic animals including rainbow trout (*Oncorhynchus mykiss*) [24], gilthead sea bream (*Sparus aurata*) [25], Japanese seabass (*Lateolabrax japonicas*) [26], yellow catfish (*Pelteobagrus fulvidraco*) [27] and grass carp (*Ctenopharyngodon idella*) [19]. In fish, previous studies found that excess dietary vegetable oil can inhibit CPT 1 gene expression, resulting in increased body fat content [28, 29].

Chinese mitten crab *Eriocheir sinensis* (Crustacea, Decapoda) is an economically important aquaculture species in China, with annual aquaculture production of ~800,000 tons in 2016 [30]. The hepatopancreas is the main location for lipid storage and metabolism in *E*. *sinensis*, which contains 30–80% lipids depending on developmental stage and gender [31]. During ovarian development, female *E*. *sinensis* accumulate lipids in the ovaries, and mature ovaries contain around 30% lipids based on dry weight [32, 33]. Therefore, *E*. *sinensis* is considered a good model for studying lipid metabolism in crustaceans [34–36]. However, information on CPT genes in crustaceans is currently limited [37, 38].

Formulated diets have been widely used in *E*. *sinensis* farming, and vegetable oil can be used to replace a proportion of fish oil in feed [36, 39]. However, information on the effects of dietary fish oil replacement on expression of CPT genes in *E*. *sinensis* has not been reported. Therefore, in the present work, the complete coding sequence (CDS) for each of the three CPT isoforms was obtained from the draft genome of *E*. *sinensis* and cloned by PCR [40]. The isoforms were subjected to molecular characterisation and tissue expression pattern analysis in male and female crabs. To investigate the effect of dietary fatty acid profile and explore the possible roles of these isoforms in fatty acid β-oxidation, we investigated the effect of dietary fish oil replacement by vegetable oil on mRNA expression levels in the hepatopancreas. The results provide fundamental molecular and expression pattern information, reveal the effects of dietary fish oil replacement, and provide a scientific basis for future research on lipid metabolism in crustaceans.

## Materials and methods

### Experimental diets and analysis

Refer to Zhao et al. [36], five isonitrogenous (39.5% crude protein) and isolipidic (14.0% crude lipid) experiment diets were formulated to replace 0, 25, 50, 75 and 100% of fish oil (Diets 1–5#, respectively) with a vegetable oil mixture of soybean oil: rapeseed oil = 1:1 (m/m). All dry ingredients in experimental diets were ground to pass through a 250 μm sieve before mixing, then combined and thoroughly mixed to homogeneity by feed mixer. The diets were extruded through a 5 mm orifice, and the lipid ingredients were added with vacuum spraying. All experimental diets were cooled and dried, then packed in sealing bags and stored at -20°C until use.

The contents of moisture, crude protein, and ash in experimental diets were analysed according to AOAC procedures [41]. Total lipids were extracted with chloroform-methanol (2:1, v/v) according to [42]. Fatty acid profiles were analysed based on the method described by [43]. The formulation, proximate composition and the fatty acid profile of five experimental diets are shown in Table 1 and Table 2.

**Table 1:** Formulation of five experimental diets.

Ingredients (%)	Diet 1#	Diet 2#	Diet 3#	Diet 4#	Diet 5#
Soybean meal	20.00	20.00	20.00	20.00	20.00
Rapeseed meal	14.54	14.54	14.54	14.54	14.54
Wheat gluten	2.00	2.00	2.00	2.00	2.00
fish meal	18.00	18.00	18.00	18.00	18.00
Wheat flour	14.00	14.00	14.00	14.00	14.00
Brewer's yeast	4.00	4.00	4.00	4.00	4.00
Squid meal	10.00	10.00	10.00	10.00	10.00
Shrimp meal	3.00	3.00	3.00	3.00	3.00
Vitamin premix ^a^	0.76	0.76	0.76	0.76	0.76
Mineral premix ^b^	1.20	1.20	1.20	1.20	1.20
Ca(H_2_PO_4_)_2_	1.50	1.50	1.50	1.50	1.50
Choline chloride (50%)	0.50	0.50	0.50	0.50	0.50
Inositol	0.30	0.30	0.30	0.30	0.30
Fish oil	8.00	6.00	4.00	2.00	0
Soybean oil	0	1.00	2.00	3.00	4.00
Rapeseed oil	0	1.00	2.00	3.00	4.00
Soy lecithin	2.00	2.00	2.00	2.00	2.00
Salt	0.20	0.20	0.20	0.20	0.20

^a^ Vitamin premix (per kg diet): vitamin A, 62,500 IU; vitamin D_3_, 15,000 IU; vitamin E, 1.05 g; vitamin K_3_, 35.4 mg; vitaminB_1_, 100 mg; vitaminB_2_, 150 mg; vitaminB_6_,150 mg; vitaminB_12_, 0.2 mg; vitamin C, 700 mg; biotin, 4 mg; D-pantothenic acid, 250 mg; folic acid, 25 mg; nicotinamide, 300 mg.

^b^ Mineral premix (per kg diets): FeSO_4_·H_2_O, 200 mg; CuSO_4_·5H_2_O, 96 mg; ZnSO_4_·H_2_O, 360 mg; MnSO_4_·H_2_O, 120 mg; MgSO_4_·H_2_O, 240 mg; KH_2_PO_4_, 4.2 g; NaH_2_PO_4_, 0.5 g; KI, 5.4 mg; CoCl_2_·6H_2_O, 2.1 mg; Na_2_SeO_3_, 3 mg.

Note that this table cited in the previously published article by Zhao et al [36].

**Table 2:** Proximate composition and fatty acid profiles of experimental diets.

Items	Diet 1#	Diet 2#	Diet 3#	Diet 4#	Diet 5#
*Proximate composition (% dry weight)*					
Moisture	11.81	11.45	11.85	12.34	11.18
Crude protein	39.55	39.22	39.38	39.59	39.55
Crude lipid	13.84	14.75	14.18	13.23	14.03
Ash	9.21	9.15	9.14	9.24	9.40
*Fatty acid profiles (% total fatty acids)*					
C14:0	4.08	3.63	2.69	1.85	0.92
C15:0	0.59	0.54	0.40	0.25	0.12
C16:0	20.73	19.39	17.09	15.02	12.61
C17:0	0.54	0.49	0.39	0.27	0.15
C18:0	4.52	4.43	4.20	3.89	3.69
C20:0	0.51	0.53	0.46	0.39	0.48
Σ SFA	31.49	29.51	25.47	22.17	18.07
C16:1	4.14	3.89	2.96	1.96	1.04
C18:1n9	15.22	18.77	24.32	28.85	31.91
C18:1n7	3.17	3.38	3.16	3.12	3.13
C20:1	0.90	0.92	0.85	0.75	0.68
Σ MUFA	24.21	27.72	31.96	34.24	37.22
C18:2n6	18.67	18.51	23.83	28.79	33.06
C20:2n6	1.26	1.20	0.87	0.61	0.37
C22:2n6	0.38	0.35	0.26	0.18	0.09
C18:3n3	2.42	2.68	3.44	4.20	5.22
C20:4n6	0.80	0.75	0.52	0.33	0.19
C20:5n3	6.26	5.80	4.25	2.90	1.56
C22:6n3	9.92	9.39	6.62	4.37	2.15
Σ PUFA	39.76	38.68	39.79	41.56	42.78
Σ n-3PUFA	18.66	17.87	14.32	11.47	8.96
Σ n-6PUFA	21.11	20.81	25.48	30.09	33.81
Σ LC-PUFA	17.03	15.94	11.39	7.60	3.87
n-3/n-6	0.88	0.86	0.56	0.38	0.27

Fatty acids contents < 0.4% are not listed in this table. SFA, saturated fatty acid; MUFA, monounsaturated fatty acid; LC-PUFA, long chain polyunsaturated fatty acid.

Note that this table cited in the previously published article by Zhao et al [36].

### Experimental setup and culture management

Experiments were conducted at Chongming mitten crab research station, Shanghai Ocean University, Shanghai, P.R. China. In late August 2014, pond-reared crabs that had completed puberty moult were obtained from outdoor earth ponds at the research station [44, 45]. The initial body weight of male and female crabs was 135-165g and 100-120g, respectively. Only healthy, active and intact crabs were selected and randomly stocked into 20 small outdoor experimental ponds (length × width × depth = 7.8 m × 7.8 m × 0.7 m) at a density of 50 crabs/pond (25 males and 25 females). Double layer nets (height = 100 cm, mesh size = 2 mm) surrounded each pond to prevent crabs from entering/leaving ponds. Smooth plastic boards (height = 35 cm, thickness = 0.5 mm) were attached to the top of each net to prevent crabs from escaping by climbing nets. The water inlet and outlet of each enclosure was covered with plastic nets (mesh size = 0.17 mm) to exclude indigenous fish and other predators from the water source and drainage channel. Alligator weed (*Alternanthera philoxeroides*) was transplanted at 1.0 m × 1.0 m intervals on the bottom of each pond to provide shelter and to help maintain water quality. Each of the five dietary treatments (Diets 1#-5#) were repeated in quadruplicate, with 50 crabs per replicate. Prior to commencement of the experiment, crabs were acclimated in the ponds for 3–5 days until initiation on the 15th September 2014 and lasted for 30 days.

Crabs were fed one of the five experimental diets once daily at 18:00 with a ration of approximately 1–3% of total biomass depending on the residual feed, water temperature and weather. Every morning at 9:00, excess feed and dead crabs were recorded. During the experimental period, a water depth of 70 cm was maintained throughout, and 30–50% of water in each pond was exchanged every 2 weeks. dissolved oxygen was maintained at ~4 mg/L, ammonia and nitrite were maintained below 0.5 mg/L and 0.15 mg/L, respectively and the pH fluctuated between 7.0 and 9.0.

### Sample collection

At termination of the feeding trial, crabs were fasted for 24 h and then were treated with cold shock method to minimize suffering before harvest. Hepatopancreas tissue from eight male crabs and eight female crabs from each treatment (two crabs from each replicate) were randomly sampled to investigate the effects of fish oil replacement on CPT gene expression. Meanwhile, 12 tissues from five male crabs (brain ganglia, thoracic ganglia, hepatopancreas, stomach, intestine, heart, gill, haemolymph, muscle, germinative area, seminal vesicle and accessory gland) and 10 tissues from five female crabs (brain ganglia, thoracic ganglia, hepatopancreas, stomach, intestine, heart, gill, haemolymph, muscle and ovary) fed Diet 1# were collected for testing of CPT tissue expression. Samples were immediately frozen in liquid nitrogen and stored at -80°C until use.

### Identification and cloning of CPT genes

Detailed searches using bioinformatics tools and available databases were employed to identify putative CPT candidate genes in the draft genome of *E*. *sinensis*. Three putative CPT isoforms (CPT1α, CPT1β and CPT2) were found and PCR-specific primers were designed to amplify full-length cDNAs (Table 3). Total RNA from *E*. *sinensis* was extracted using RNAiso Plus reagent (9109; TaKaRa Bio, Japan) according to the manufacturer’s protocol, and stored at -80°C. RNA concentration was measured using a Q5000 ultraviolet spectrophotometer (Quawell Q5000, USA), and RNA integrity was evaluated by 1% agarose gel electrophoresis. First-strand cDNAs were synthesised using a reverse first-strand cDNA synthesis kit (RR036A; TaKaRa). PCR amplification was conducted at 95°C for 5 min followed by 35 cycles of 95°C for 30 s, 55°C for 30 s, 72°C for 2 min, and a final extension of 72°C for 10 min. Amplified products of the expected size were gel-purified using a TIANgel Midi Purification Kit (DP209; TIANGEN, China), cloned into the pMD19-T vector (6013A; TaKaRa), and the resulting construct containing the target DNA was transformed into *Escherichia coli* DH5α cells. Positive clones were selected by blue-white spot screening and confirmed by DNA sequencing.

**Table 3:** Specific primers used for CDS cloning and qRT-PCR of CPT family members from *E*. *sinensis*.

Primers	Sequences (5′-3′)	Size (bp)	Usage
CPT1α-F	ATGAACACGCACTCCTTCCCAC	2748	CDS cloning
CPT1α-R	CTACGAGTCAAGTTTGGAGTCGTC		CDS cloning
CPT1β-F	ATGGCGGAGGCTCACTCAG	2328	CDS cloning
CPT1β-R	TTATTTCTTGGCCAGTCCAGC		CDS cloning
CPT2-F	ATGAGAGCTGATAGGAAAAATAAA	2052	CDS cloning
CPT2-R	TTAAGAGGTTGAGAGCACGTC		CDS cloning
CPT1α-F	TGTTGAAGCCTGACCTTCCA	174	qRT-PCR
CPT1α-R	GGTTGTAGCAGCAGCCATAC		qRT-PCR
CPT1β-F	GCCTCTGATGGATGACGAGA	160	qRT-PCR
CPT1β-R	TCCTCGCAGGTACACAAACT		qRT-PCR
CPT2-F	TGGCTATGCAGACCCTTTGA	157	qRT-PCR
CPT2-R	GGTCCATCCCGTAACTTTGC		qRT-PCR
β-actin-F	GCATCCACGAGACCACTTACA	266	qRT-PCR
β-actin-R	CTCCTGCTTGCTGATCCACATC		qRT-PCR

### Sequence and phylogenetic analysis

Sequences were verified and analysed using the NCBI BLAST tool (http://blast.ncbi.nlm.nih.gov/). Transmembrane domains of proteins were predicted using TMHMM server v.2.0 (http://www.cbs.dtu.dk/services/TMHMM). Signal peptides were predicted with SignalP v.4.1 (http://www.cbs.dtu.dk/services/SignalP/). CPT gene family members were predicted using the conserved domains profile (http://smart.embl-heidelberg.de/). Isoelectric point (pI) and molecular weight were calculated using ProtParam within the ExPASy server (http://web.expasy.org/protparam/). Multiple protein sequence alignment was performed using CrustalW program, and phylogenetic trees were constructed by Bayesian inference with MEGA 6.0 [46].

### Quantitative real-time PCR (qRT-PCR)

qRT-PCR was applied to investigate the tissue distribution and CPT mRNA levels in the hepatopancreas in response to changes in dietary fish oil. Extraction and purification of total RNA from each sample and first-strand cDNA synthesis were performed as described above. All real-time reactions were performed on a FAST-7500 system (ABI-7500; ThermoFisher, Singapore) using a SYBR RT-PCR Kit (RR420A, TaKaRa). The total reaction volume was 10 μL containing 5 μL of 2× SYBR Master Mix, 1 μL of diluted cDNA, 0.2 μL (10 mM) of forward and reverse primers and ROX Reference Dye II, and 3.4 μL of Rnase-free dH_2_O. The reaction conditions consisted of a 30 s initial denaturation at 95°C, followed by 40 cycles of 5 s at 95°C, 34 s at 60°C, and 30 s at 72°C. All samples were analysed in triplicate and normalised against the expression of the β-actin internal reference gene. Primers used for qRT-PCR are listed in Table 3. Data were averaged by three technical replicates in the same run, and results are presented as the means ± standard error calculated using the 2^−ΔΔCt^ method [47].

### Statistical analysis

Statistical analysis was performed using SPSS 18.0 (Chicago, USA). Results are presented as mean ± standard error (SE). Statistical significance was determined using one-way analysis of variance (ANOVA) and post-hoc Duncan’s multiple range tests (*P* < 0.05 was considered statistically significant).

## Results

### Sequence analysis

In the present study, the CDS was obtained for of each of the three CPT isoforms of *E*. *sinensis*, which were named CPT 1α (GenBank accession no. MH037158), CPT 1β (GenBank accession no. MH037159) and CPT 2 (GenBank accession no. MH037160). Sequence analysis revealed that CPT 1α, CPT 1β and CPT 2 have a full-length CDS of 2748, 2328 and 2052 bp, encoding polypeptides of 915, 775 and 683 amino acid residues, with a theoretical pI of 9.07, 8.79 and 7.93 and a molecular weight of 104.98, 88.25 and 76.29 kDa, respectively. The instability index (II) of the CPT 1β protein was computed to be 38.37, which classifies the protein as stable. However, the instability index (II) of the CPT 1α and CPT 2 proteins were computed to be 51.03 and 41.58, respectively, which classifies them as unstable.

**Fig 1:**
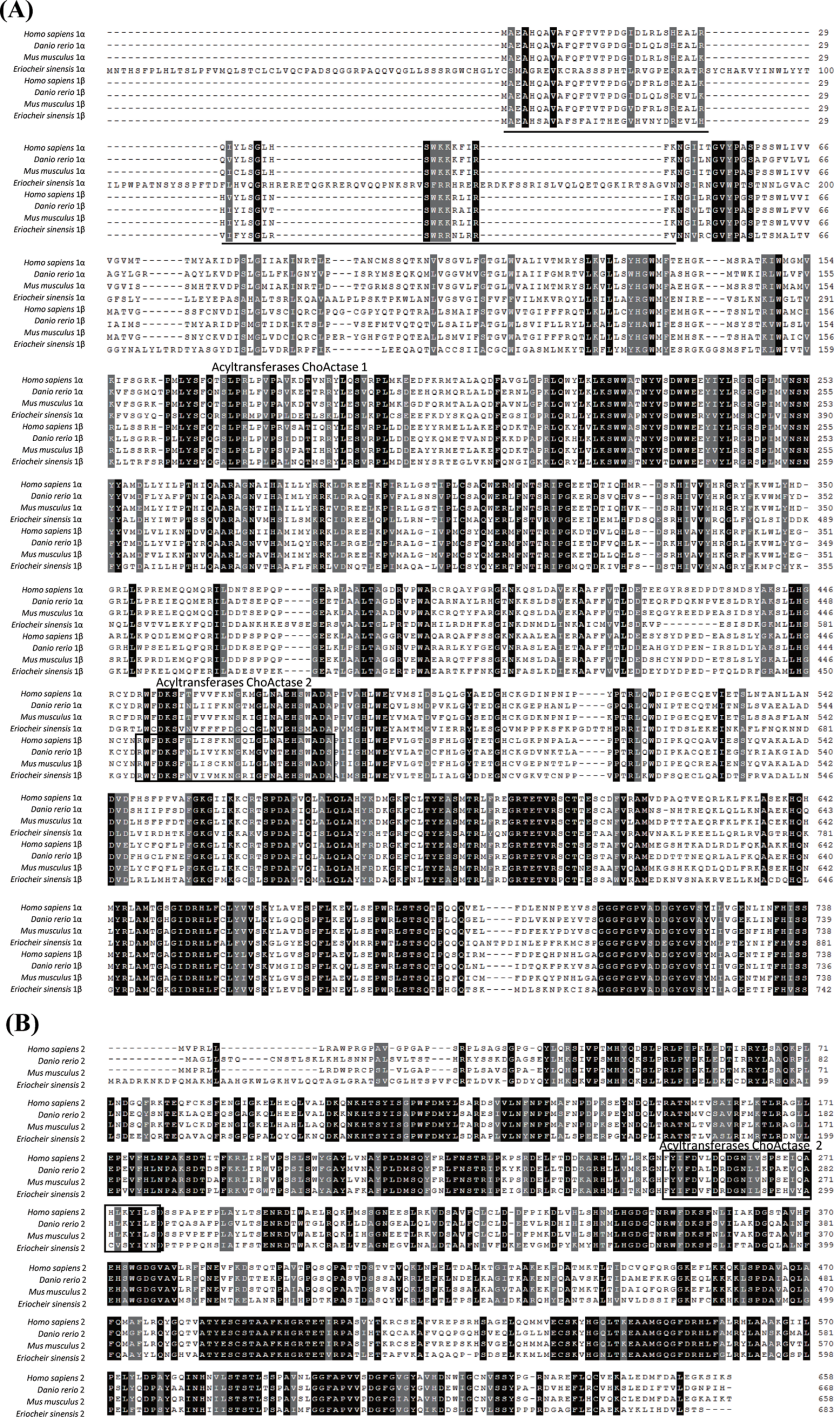
**Alignment of the deduced amino acid sequences of carnitine palmitoyltransferase 1 (CPT 1; A) and CPT2 (B) from *Homo sapiens*, *Danio rerio*, *Mus musculus* and *Eriocheir sinensis*.** Identical residues are shaded dark grey, and similar residues are shaded grey. CPT family proteins are predicted to include two key domains; the CPT N-terminal domain is underlined, and acyltransferase choActase domains 1 and 2 are boxed.

Fig 1 shows the deduced amino acid sequences of the three CPT genes. There are two transmembrane regions in CPT 1α and CPT 1β, but no transmembrane in CPT2. A CPT N-terminal domain and two acyltransferase choActase domains are present in CPT 1β, but CPT 1α lacks these domains, and only one acyltransferase choActase domain is present in CPT 2.

### Multiple sequence alignment and phylogenetic analysis

The identities of CPT family members and the other representative CPT amino acid sequences were investigated via multiple sequence alignment using Clustal W (Fig 1). Sequence alignment CPT 1α, CPT 1β and CPT 2 revealed high sequence homology with CPT proteins from other species, with most amino acids conserved.

**Fig 2:**
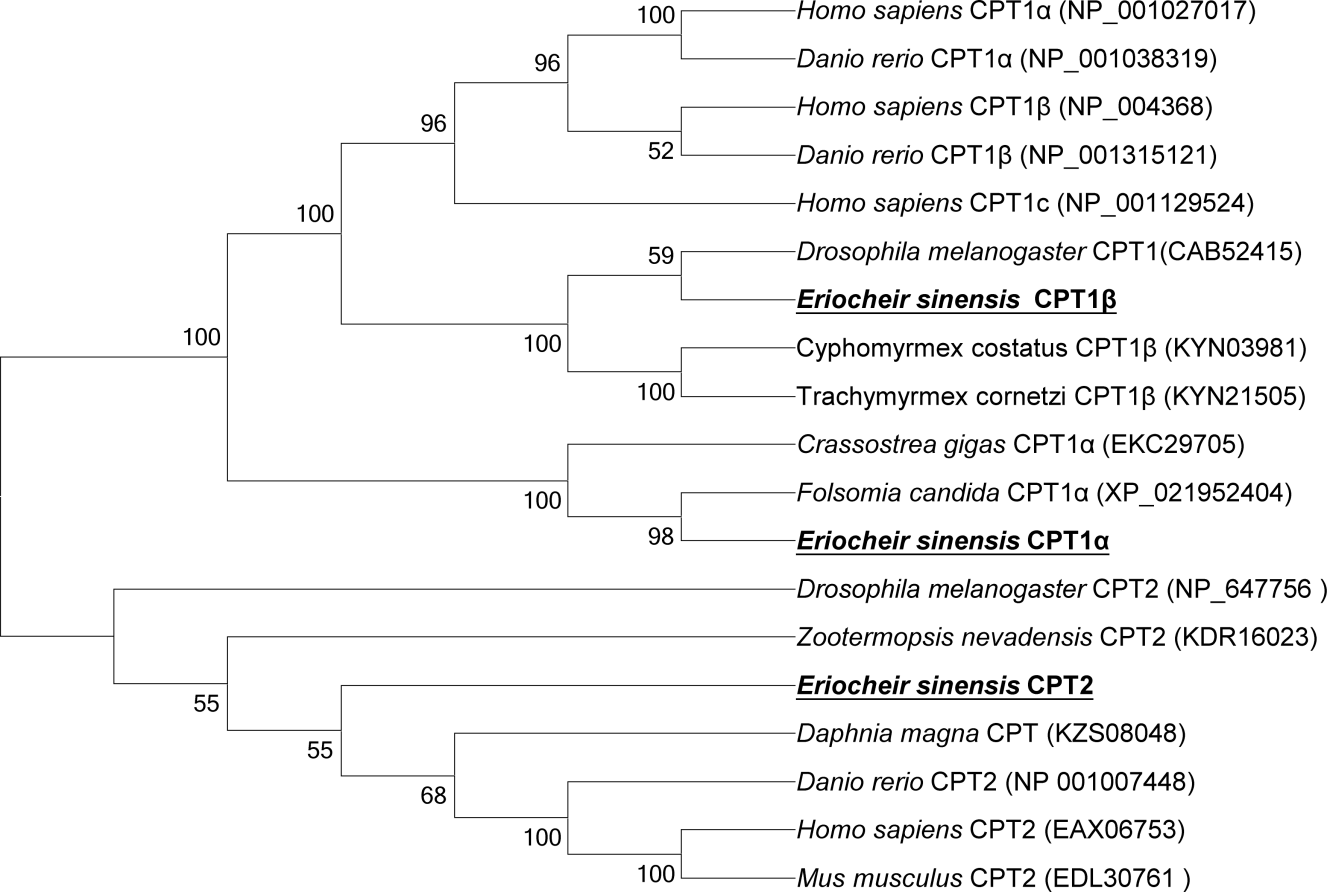
Phylogenetic tree comparing the deduced amino acid sequences of *E*. *sinensis* CPT family members with homologs from various organisms. The tree was constructed using the neighbour-joining method with MEGA 6.0, and bootstrap values supporting branch points are expressed as the percentage of 1000 replicates.

A neighbour-joining phylogenetic tree was constructed based on CPT amino acid sequences using MEGA 6.0 software, with confidence in the resulting tree branch topology measured by bootstrapping through 1000 pseudo replicates. CPT 1α and CPT 1β of *E*. *sinensis* clustered together with CPT 1 from other species (vertebrates and invertebrates), separate from the CPT 2 cluster. In addition, CPT 1α and CPT 1β clustered most closely to *Folsomia candida* CPT 1α (58% identity) and *Drosophilla melanogaster* CPT 1 (60% identity). CPT 2 formed one monophyletic sub-branch, and clustered with other CPT2 proteins (Fig 2).

### Tissue expression patterns

**Fig 3:**
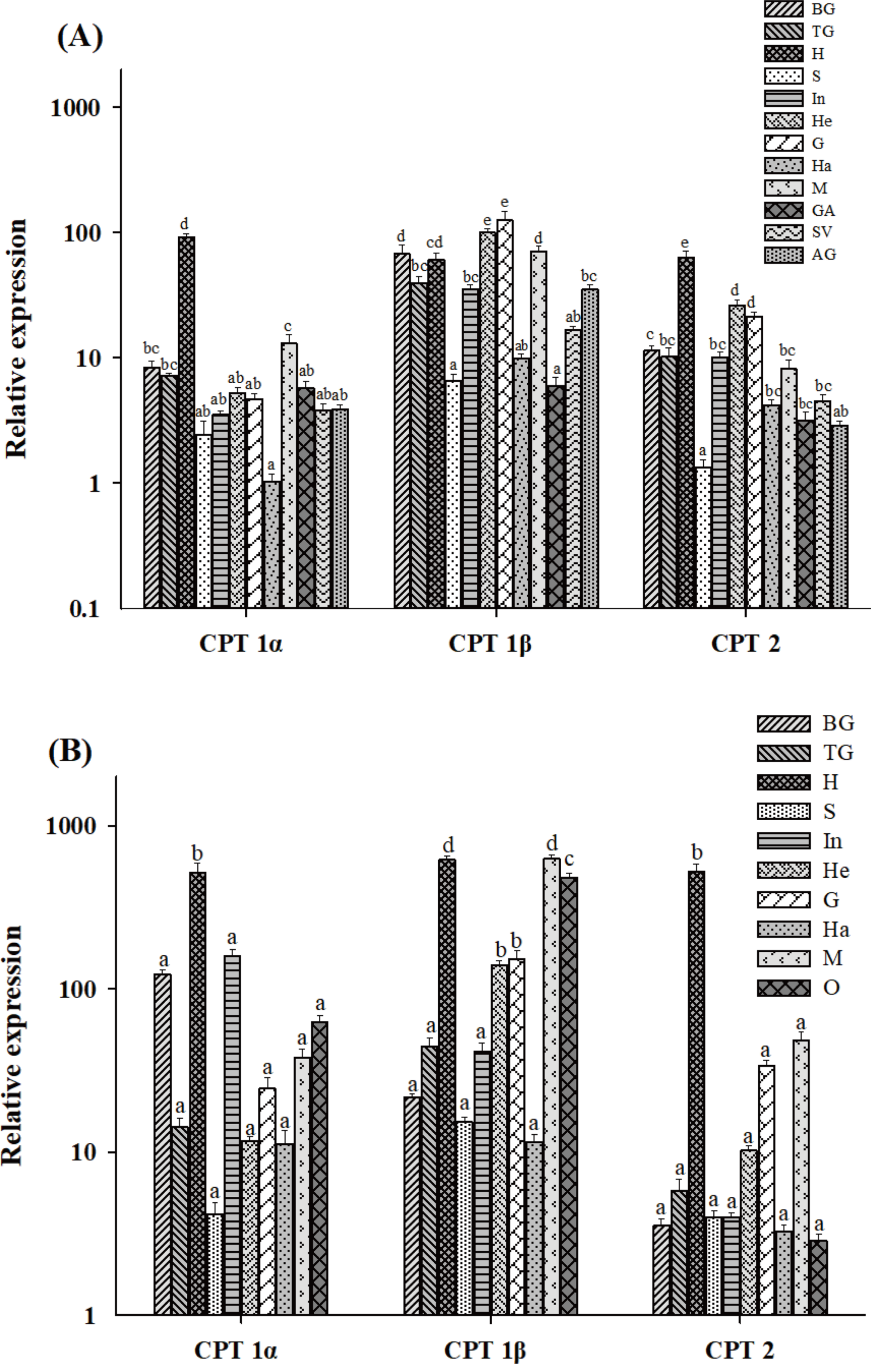
Tissue expression levels of CPT family members. Total RNA is normalized against the β-actin internal reference gene in brain ganglia (BG), thoracic ganglia (TG), hepatopancreas (H), stomach (S), intestine (In), heart (He), gill (G), haemolymph (Ha), muscle (M), germinative area (GA), seminal vesicle (SV), accessory gland (AG) and ovary (O) in male (A) and female (B) *E*. *sinensis*. Values are means ± SE (n = 5). Bars that do not share a common letter (a, b, c, d or e) indicate significant differences among different tissues (*P* < 0.05).

All three CPT isoforms were expressed in all tissues tested, including brain ganglia, thoracic ganglia, hepatopancreas, stomach, intestine, heart, gill, haemolymph, muscle, germinative area (males), seminal vesicle (males), accessory gland (males) and ovary (females; Fig 3). Expression levels varied among isoforms and tissues (Fig 3A). In male crabs, the highest CPT 1α mRNA levels were detected in the hepatopancreas, while the accessory gland had the lowest CPT 1α mRNA levels (*P* < 0.05). By contrast, CPT 1β expression was highest in gill and heart, followed by muscle, brain ganglia and hepatopancreas. For CPT 2, expression levels were ordered hepatopancreas > heart > gill > brain ganglia > thoracic ganglia > seminal vesicle > intestine > haemolymph > germinative area > accessory gland. CPT 1β mRNA expression was generally higher than CPT 1α and CPT 2 expression in the same tissue, except in the hepatopancreas. CPT 1α and CPT 2 displayed similar expression patterns among the tested tissues.

In female crabs (Fig 3B), CPT 1α was mainly expressed in the hepatopancreas, followed by the intestine, and expression was lowest in the stomach. Expression of CPT 1β was highest in muscle, followed by hepatopancreas and ovary, and lowest in haemolymph. CPT 1β mRNA expression was the highest among the three isoforms in most tissues, similar to male crabs. CPT 2 expression was highest in the hepatopancreas, but no significant differences were observed among the other tissues, although expression levels in most tissues were generally lower than those of the other two isoforms.

### Effect of dietary fish oil replacement on CPT gene expression in the hepatopancreas

**Fig 4:**
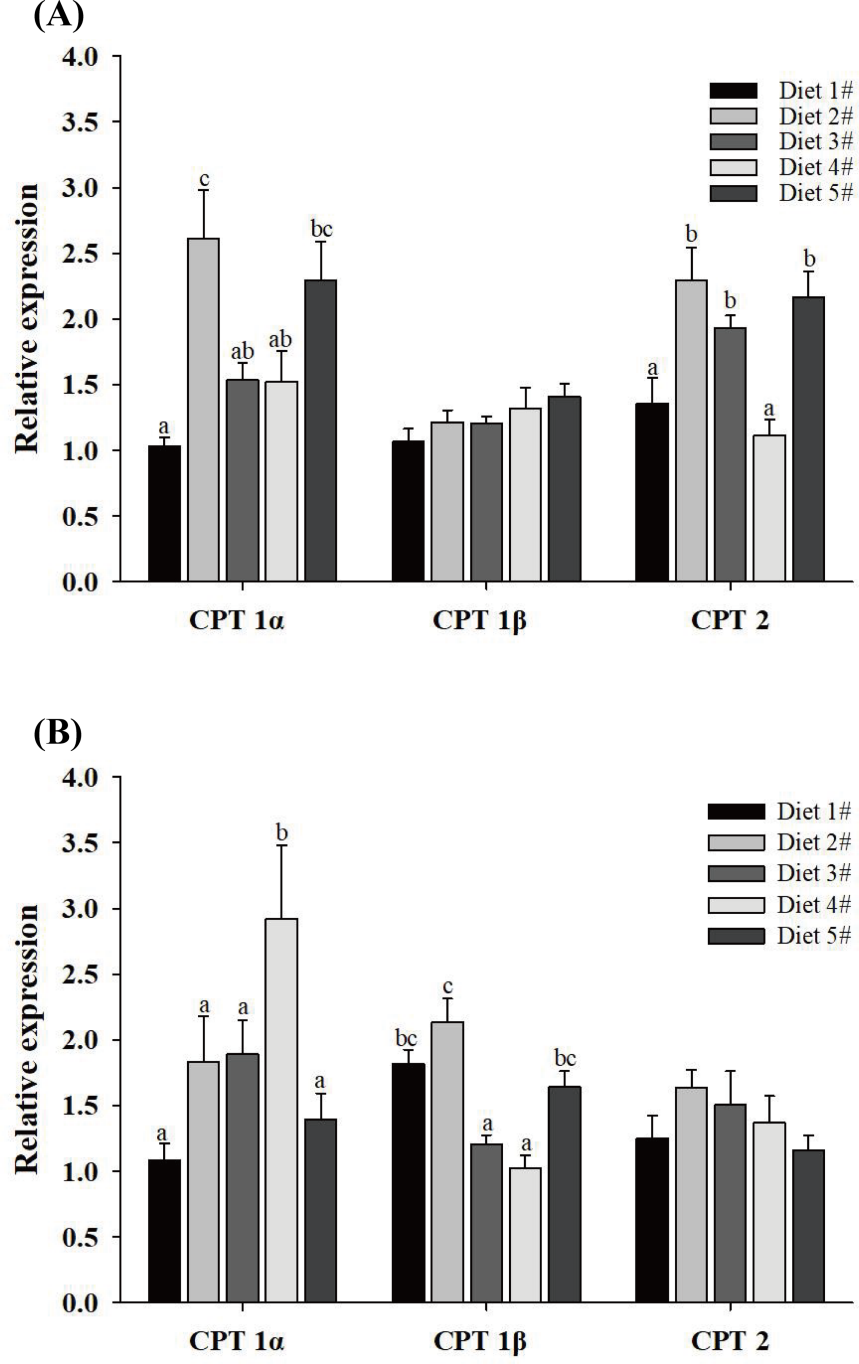
**Effects of replacing dietary fish oil with vegetable oil on CPT isoform mRNA expression levels in the hepatopancreas of male (A) and female (B) *E*. *sinensis*.** Data (means ± SE, n = 8) were normalized against the β-actin gene. Bars with different letters indicate statistical significance (*P* < 0.05).

CPT mRNA expression levels of the three isoforms in the hepatopancreas following the five dietary treatments are shown in Fig 4 for males and females. Overall, in male cabs (Fig 4A), dietary fish oil replacement had a significant effect on CPT 1α and CPT 2 expression, and a similar trend was found for both genes. With increasing dietary vegetable oil replacement, CPT 1α and CPT 2 mRNA levels first increased significantly, then decreased up to Diet 4#, and Diets 2# and 5# generally resulted in the highest CPT 1α and CPT 2 mRNA expression levels (*P* < 0.05). Although the expression level of CPT 1β tended to increase with increasing replacement of dietary fish oil, no significant differences were found among the five treatments (*P* > 0.05)

For female crabs (Fig 4B), mRNA expression levels of the three CPT genes in the hepatopancreas increased then declined with increasing dietary fish oil replacement. Compared with the other treatments, Diet 4# most significantly upregulated the mRNA levels of CPT 1α (*P* < 0.05). Expression of CPT 1β in Diet 2# was significantly higher than in Diets 3#-5# (*P* < 0.05). CPT 2 was expressed most highly in Diet 2#, but there were no significant differences among the five treatments (*P* > 0.05).

## Discussion

Members of the CPT family play an important role in the control and regulation of fatty acid β-oxidation [3]. In mammals, there are three CPT 1 isoforms resulting from genome duplication early in vertebrate evolution [8–10]. In an early study, Gutières et al. reported that at least one CPT 1 gene is present in trout [24]. More recently, different isoforms of CPT genes have been discovered via the catalytic examination of CPT 1 activity, and diverse tissue distributions of expressed CPT genes have been reported in aquatic fish [25, 48–50]. Five CPT 1 isoforms have now been identified in rainbow trout (CPT 1α1a, CPT 1α1b, CPT 1α2, CPT 1β1a and CPT 1β1b) [49], while seven isoforms (CPT 1α1a-1a, CPT 1α1a-1b, CPT 1α1a-1c, CPT 1α1a-2, CPT 1α2a, CPT 1α2b-1a and CPT 1β) have been identified in *Synechogobius hasta* [51], and five isoforms (CPT 1α1a, CPT 1α1b, CPT 1α2a, CPT 1α2b and CPT 1β) have been reported for *Ctenopharyngodon idellus* [19]. Sequence data for CPT 1 is now available for *Danio rerio*, *Sparus aurata*, *Lates japonicas* and fish others [25, 26, 49]. By contrast, CPT 2 has been found in fewer aquatic species [19], and cDNA sequences for all CPT genes in aquatic crustaceans are limited.

In the present study, we successfully identified and characterised three CPT isoforms from *E*. *sinensis* (CPT 1α, CPT 1β and CPT 2). Interestingly, duplication of CPT 1α was not observed, and CPT 1γ was not present. The deduced polypeptides share high homology with CPT proteins from other species. Moreover, prediction of protein domains suggests that CPT 1β and CPT 2 in *E*. *sinensis* include an acyltransferase choActase domain. The N-terminal domain (residues 1–150) of mammalian CPT 1 contains mitochondrial targeting information and has been implicated in the insertion of the protein in the outer mitochondrial membrane [52]. Our current results suggest that the elements responsible for membrane insertion are also conserved in *E*. *sinensis* CPT 1β. However, CPT 2 in *E*. *sinensis* has no N-terminal domain, which is important for sensitivity to malonyl-CoA [5], suggesting that CPT 2 might not be essential for mitochondrial fatty acid uptake in this species. Intriguingly, CPT 1α also lacks an N-terminal domain, indicating that there might be differences between *E*. *sinensis* and other fish and mammal species. In previous studies, CPT 1 reported to include two transmembrane domains most fish and mammals [25, 52, 53]. In the present study, hydrophobicity analysis of the three CPT isoforms predicted the presence of two transmembrane domains in CPT 1α and CPT 1β, but nor transmembrane helices in CPT 2. However, the phylogenetic tree based on the sequence alignment strongly suggests that the three *E*. *sinensis* isoforms belong to the CPT family.

β-oxidation of fatty acids is a complex pathway in mammals, and the activity of CPT enzymes is controlled by various regulatory mechanisms including transcriptional regulation, physiological regulation by malonyl-CoA, and pharmaceutical regulation [14]. CPT 1 is controlled by the steady state level of malonyl-CoA since it is sensitive to inhibition by his compound [54]. The sensitivity of CPT 1α to malonyl-CoA inhibition is increased by insulin and decreased by thyroid hormone, while the sensitivity of CPT 1β responds to malonyl-CoA fluctuations produced by changes in fuel supply, such as glucose, fatty acids, lactate/pyruvate and ketone bodies, suggesting that CPT 1β is regulated by fuel, while CPT1α is hormonally regulated [55, 56]. Sensitivity of CPT 1 to malonyl-CoA is also influenced by phosphorylation, as well as changes in the lipid environment [57–59]. Peroxisome proliferator-activated receptors (PPARs) can also regulate the expression of genes encoding enzymes involved in fatty acid β-oxidation, including CPT enzymes [60]. Barrero et al. found that expression of CPT2 was upregulated significantly in response to a PPARα agonist in a PPARα-dependent manner in heart and liver [61]. It is noteworthy that the relationship between fatty acid oxidation and CPT levels, and the sensitivity of CPT1 to malonyl-CoA, appears to be similar in fish and mammals [13]. Therefore, the involvement of the three CPT isoforms of *E*. *sinensis* in malonyl-CoA sensitivity should be investigated in future studies.

The tissue distribution profiles of CPT isoforms in *E*. *sinensis* were determined to evaluate their physiological roles. In general, mRNA expression of all three CPT isoforms was detected in all tested tissues, albeit at varying levels (Fig 3). CPT 1α, CPT 1β and CPT 1γ are expressed highly in liver, muscle, and brain, respectively, in mammals [8–10]. The midgut gland of the freshwater prawn *Macrobrachium borellii* is a highly active site for lipid metabolism, and it has a high capacity for using saturated long-chain fatty acids via catalysis by CPT 1 [37]. In blunt snout bream (*Megalobrama amblycephala*), the highest CPT 1α mRNA levels occur in heart and white muscle [53], and in most fish species, expression is abundant in liver [19, 27]. Similarly, CPT 1α mRNA levels in the hepatopancreas of both male and female *E*. *sinensis* were higher than in other tissues. The hepatopancreas is a major site for lipolysis, and our results suggest that CPT 1α might be a major player in the degradation of fatty acids in *E*. *sinensis*. CPT 1β was predominantly expressed in gill, heart, muscle and hepatopancreas in males, and in muscle, hepatopancreas and ovary tissue in females, with very low expression in other tissues. This distribution is in agreement with previous results for CPT 1β in *S*. *aurata*, *P*. *fulvidraco* and *C*. *idella* [19, 25, 27]. Furthermore, higher expression of CPT 1β in ovaries suggests that fatty acid β-oxidation also occurs in female gonads. The tissue mRNA expression distribution of CPT 1 also further indicates that CPT 1α is a liver isoform, while CPT 1β is orthologous to the mammalian muscle isoform. CPT 2 mRNA levels were highest in heart tissue in grass carp [19]. In *E*. *sinensis*, CPT 2 was abundantly expressed in the hepatopancreas in both males and females, similar to CPT 1α. High expression of all three CPT isoforms in the hepatopancreas of *E*. *sinensis* further suggests that it is a major site of lipid metabolism in Crustacea and indicates differences in lipid metabolism between males and females.

Regulation of CPT gene expression has received considerable interest to improve our better understanding of the regulation of fatty acid β-oxidation in commercial aquaculture species. Fish oil (FO) produced by wild fisheries is a major lipid resource in aquatic feed. However, due to increasing costs and limited supplies of global FO [62, 63], it cannot meet the demands of the aquaculture and aquatic feed industries. Thus, identification of other suitable lipid resources for substituting FO in fish and crustacean diets is much needed [64, 65]. Vegetable oils such as soybean, linseed and rapeseed oil are promising candidates for FO replacement due to steadily increasing production, high availability and better economic value [66, 67]. Furthermore, linoleic acid and linolenic acid, which are rich in vegetable oils, can be partially converted by aquatic animals to LC-PUFAs such as arachidonic acid (ARA), eicosapentaenoic acid (EPA) and docosahexaenoic acid (DHA) [33]. Aquatic animals can also use these fatty acids for oxidation. Therefore, vegetable oils are widely used in aquatic feed. Since CPTs play an important role in the oxidation of LC-FAs, replacement of FO in feed may have a significant effect on CPT gene expression in aquatic animals. In the present study, we investigated the effects of dietary FO replacement by blending vegetable oils on mRNA expression of CPT family members in the hepatopancreas of *E*. *sinensis*.

In mammals, long-chain fatty acids (16:0, 18:1n9, 18:2n6, 20:5n3 and 22:6n3) can significantly increase CPT 1 mRNA levels compared with medium-chain fatty acids (8:0 and 10:0), but they have no effect on CPT 2 mRNA expression [68]. In male *E*. *sinensis*, the results of the present study showed that CPT 1α and CPT 2 expression levels in the hepatopancreas first increased then decreased with increasing dietary FO replacement. This may be due to a significant increase in dietary C18:1n9 and C18:2n6 levels in Diet 2#, since some of these LC-FAs may be used for energy provision via CPT 1α and CPT 2. An increase in dietary vegetable oils can lead to high lipid deposition in the hepatopancreas [69]. This suggests excessive dietary C18:1n9 and C18:2n6 intake may decrease hepatopancreatic CPT 1α and CPT 2 mRNA expression. However, Diet 5# yielded higher CPT1α and CPT 2 mRNA levels than Diets 3# and 4#, possibly due to unbalanced dietary fatty acids (e.g. low dietary 20:5n3 and 22:6n3, and high 18:1n9, 18:3n3 and 18:2n6). We speculate that the apparent differences in the effects of different fatty acids on CPT2 expression in mammals and *E*. *sinensis* may be due to the different species. However, expression of CPT 1β tended to increase with increasing FO replacement, but no significant differences were found, which suggests that replacement of dietary FO made no difference to CPT 1β mRNA expression. This result further indicates that CPT 1β might principally regulate fatty acid oxidation in muscle in male *E*. *sinensis*.

In female *E*. *sinensis*, the highest CPT 1α and CPT 2 expression levels were detected in the hepatopancreas of animals fed Diets 4# and 2#, respectively, and expression was decreased following consumption of Diet 5#. A similar result was observed in a previous study on *E*. *sinensis* in which CPT 1 mRNA levels were significantly increased in the 1:1 fish oil: linseed oil (FLO) group relative to the FO group [70]. Several other studies also suggest that a diet supplemented with vegetable oil increases CPT 1 expression [29, 71]. In the present study, CPT 1α and CPT 2 expression levels in the hepatopancreas first increased then decreased with increasing FO replacement. This may be due to a significant increase in C18:1n9 and C18:2n6 levels in feeds with greater replacement of dietary FO by vegetable oil. A proportion of C18 fatty acids in dietary vegetable oils may be converted to long chain polyunsaturated fatty acids (LC-PUFAs) and used for fatty acid β-oxidation to increase CPT mRNA expression [68, 72]. The decline could be related to rapid ovarian development, since large quantities of fatty acids such as 18:1n9, 18:2n6, EPA and DHA are transported to developing ovaries, leading to less β-oxidation of these fatty acids, and lower CPT 1α and CPT 2 mRNA expression levels in Diets 3#-5#. Interestingly, hepatopancreatic CPT 1β mRNA expression differed between males and females, but the reason is unclear at present, and future studies should be conducted to determine the exact roles of CPTs and their responses to different fatty acids.

## Conclusions

In summary, three CPT isoforms (CPT 1α, CPT 1β and CPT 2) were identified and characterised in *E*. *sinensis*. CPT 1α and CPT 1β have two transmembrane domains, while CPT2 has none. All three CPTs are highly expressed in the hepatopancreas in both males and females, and CPT 1β is also highly expressed in male gill, heart and muscle, and female ovaries and muscle. Dietary fish oil replacement had a significant effect on mRNA levels in the hepatopancreas for all three CPT isoforms. These results indicate that the CPT isoforms may engage in variedly physiological roles related to fatty acid β-oxidation in *E*. *sinensis*, but the underlying molecular mechanisms need to be clarified in future studies.
